# Network Pharmacology-Based Analysis of Gegenqinlian Decoction Regulating Intestinal Microbial Activity for the Treatment of Diarrhea

**DOI:** 10.1155/2021/5520015

**Published:** 2021-07-26

**Authors:** Xiaoya Li, Chenyang Zhang, Zhoujin Tan, Jiali Yuan

**Affiliations:** ^1^Hunan University of Chinese Medicine, Changsha, Hunan 410208, China; ^2^Provincial Innovation Team of Yunnan University of Chinese Medicine for Traditional Chinese Medicine to Regulate Human Microecology, Kunming, Yunnan 650500, China

## Abstract

Gegenqinlian decoction (GD) has been extensively used for the treatment of diarrhea with intestinal dampness-heat syndrome (IDHS) with a satisfying therapeutic effect. The purpose of this study is to clarify the active ingredients and mechanism of GD in the treatment of diarrhea with IDHS. The TCMSP database was used to screen out the active ingredients of the four Chinese herbal medicines in GD, and the targets of the active ingredients were predicted. We selected the targets related to diarrhea through the DisGeNET database, then used the NCBI database to screen out related targets of lactase and sucrase, and constructed the visual network to search for the active ingredients of GD in the treatment of diarrhea and related mechanisms of the targets. Combined with network pharmacology, we screened out 146 active ingredients in GD corresponding to 252 ingredient targets, combined with 328 disease targets in diarrhea, and obtained 12 lactase targets and 11 sucrase targets. The key active ingredients involved quercetin, formononetin, *β*-sitosterol kaempferol, and wogonin. Furthermore, molecular docking showed that these five potential active ingredients had good affinities with the core targets PTGS2. The active ingredients in GD (such as quercetin, formononetin, and *β*-sitosterol) may increase the microbial activity of the intestinal mucosa of mice and reduce the microbial activity of the intestinal contents through multiple targets, thereby achieving the effect of treating diarrhea.

## 1. Introduction

Syndrome research has always been the core content of traditional Chinese medicine (TCM) academic research. Research on the nature of syndromes and animal models of syndromes plays a beneficial role in revealing the scientific connotation of syndromes and the objectification of syndromes. Diarrhea with intestinal damp heat syndrome (IDHS) refers to the accumulation of dampness-heat in the intestinal tract, which is mainly manifested by abdominal pain, urgency of diarrhea, and yellowish brown and stinky feces [1]. With the changes of people's lifestyle and diet structure, IDHS diarrhea has become a multiple disease. Diabetes, chronic liver disease, kidney disease, ulcerative colitis, and other diseases are often accompanied by IDHS diarrhea. Therefore, strengthening the study of diarrhea with IDHS can expand the prevention and treatment of these major diseases.

Intestinal microorganisms are closely related to the physiology, pathology, and treatment of diseases of the body [2–4]. These can secrete a variety of enzymes, participate in the decomposition and transformation of food in the intestine, and provide energy for the growth and development of the body [5]. The occurrence of diarrhea with IDHS is often accompanied by intestinal microbial imbalance, which is bound to affect intestinal enzyme activity [6]. It was found that the activity of lactase in the intestinal tract of diarrhea irritable bowel syndrome rats was significantly lower than that of the control group (*p* < 0.05), and the activity of sucrase in the intestinal contents and mucosa of mice with dysbacteriosis and diarrhea was significantly decreased compared with the control group (*p* < 0.01) [7, 8]. Different kinds of enzymes reflect the active degree of microbial participation in biochemical reactions, while microbial activity often represents the overall activity of hydrolases and can be used as an important indicator of microbial decomposition ability [9, 10]. As a colorless nonpolar organic compound, fluorescein diacetic acid (FDA) can be hydrolyzed by nonspecific enzymes (esterase, protease, lipase, etc.) in bacteria and fungi, and its hydrolytic activity is directly proportional to the number of microbial populations [11]. Therefore, the total activity of microorganisms in the animal intestinal tract can be reflected by measuring the degree of FDA hydrolysis of intestinal samples.

Gegenqinlian decoction (GD), as a classic prescription for diarrhea with IDHS, is composed of *Puerarie Lobatae Radix* (*Pueraria lobata* (Willd.) Ohwi), *Scutellariae Radix* (*Scutellaria baicalensis* Georgi), *Coptidis Rhizoma* (*Coptis chinensis* Franch), and *Glycyrrhiza Radix Et Rhizoma* (*Glycyrrhiza uralensis* Fisch). It is better for clearing away heat and dampness, distributing benefits, and stopping diarrhea. GD is widely used in the treatment of diabetes, hypertension, hyperlipidemia, obesity, and other diseases, with significant effects [12–14]. Modern pharmacological studies have indicated that GD has antipyretic, anti-inflammatory, antibacterial, and immunological improvement and intestinal microbial regulation functions [15–17]. Oral drugs enter the small intestine through the upper digestive tract and are digested and absorbed, which play an effective role by inhibiting or inducing the enzyme activity in the intestine [18]. Therefore, exploring the effect of the active ingredients in GD on the regulation of intestinal enzyme activity in diarrhea with IDHS has become the focus of this study.

As an emerging science based on a multilayered network of disease-gene-drug, network pharmacology has been widely used to screen active ingredients, explain the overall action mechanisms, and study the pathogenesis of diseases. While the multiple ingredients of TCM have curative effects on multiple targets of diseases, network pharmacology can visualize, systematize, and inform the process principles of TCM in treating diseases [19]. A growing body of evidence suggested that the holistic concept of TCM had many similarities with the core ideas of the emerging network pharmacology and was more often applied in the treatment of complex diseases, which provided a new research strategy for the development of network pharmacology of TCM [20]. Based on these, synthesized with the previous experimental results of our research group, the effect of GD on the intestinal microbial activity of diarrhea with IDHS mice was explored to clarify the microecological mechanism of its therapeutic effect [21]. Through the overall process of screening active ingredients, network construction, and analysis, GD-active ingredients and candidate targets were predicted, and the pharmacodynamic substances and targets of GD in the treatment of diarrhea were sought, so as to provide basis for the diagnosis and treatment of syndrome differentiation in clinical diarrhea and the study on the therapeutic mechanism of TCM prescriptions.

## 2. Materials and Methods

### 2.1. Collection of Active Ingredients and Targets of GD

We input *Puerarie Lobatae Radix* (PLR), *Scutellariae Radix* (SR), *Coptidis Rhizoma* (CR), and *Glycyrrhizae Radix Et Rhizoma* (GRER) into the Traditional Chinese Medicine Systems Pharmacology (TCMSP, http://tcmspw.com/index.php) database [22] to retrieve the Chinese herbal medicine ingredients, setting oral bioavailability (OB) ≥ 30% and drug-likeness (DL) ≥ 0.18 for further screening that will meet the conditions of TCM composition as the active ingredients. Then, we searched the targets of the active ingredients through the TCMSP database and entered the obtained protein name into the Universal Protein (UniProt, https://www.uniprot.org/) database [23] to find the corresponding gene name of each target to facilitate subsequent analysis.

### 2.2. Prediction of Potential Targets for Diarrhea

Potential genes associated with diarrhea were collected from DisGeNET (https://www.disgenet.org/) [24]. For filtering the targets, “diarrhea” was defined as the key word and results were restricted to “*Homo sapiens*.” After integration of search-derived target data and elimination of the repeated genes, the therapeutic target database was obtained.

### 2.3. Construction of the Diarrhea-Potential Target-Enzyme Network

The lactase and sucrase were input in the National Center for Biotechnology Information (NCBI (https://www.ncbi.nlm.nih.gov/) database [25] to retrieve related targets, and the source of species was set for “*Homo sapiens*” for further screening. Then, we imported the potential targets of the disease and lactase and sucrase into Cytoscape 3.7.2 software to construct a network diagram of diarrhea-potential targets-enzymes.

### 2.4. Construction of the GD-Potential Active Ingredient-Potential Target-Enzyme Network

The lactase and sucrase were input in the NCBI database (https://www.ncbi.nlm.nih.gov/) [25] to retrieve related targets, and the source of species was set for “*Homo sapiens*” for further screening. Then, we imported the GD-potential targets and their corresponding active ingredients and lactase and sucrase into Cytoscape 3.7.2 software to construct a network diagram of GD-potential active ingredients-potential targets-enzymes.

### 2.5. Construction of the GD-Potential Active Ingredient-Enzyme-Diarrhea Network

We import the GD-potential targets and their corresponding active ingredients, the disease targets, and the potential targets of lactase and sucrase into the Cytoscape 3.7.2 software [26], constructing the GD-potential active ingredient-enzyme-diarrhea network diagram.

### 2.6. Molecular Docking

Molecular docking verification of key active ingredients and key targets in GD-potential active ingredients-enzymes-diarrhea was carried out. The 3D structure of the key potential target was downloaded from the Protein Data Bank [27] (PDB, http://www.RCSB.org/) database. We used AutoDock Tools software to remove water, separate ligands and hydrogenate, and calculate charge for the target proteins and used it as the receptor macromolecules. Then, we downloaded the structure diagram of the potential active ingredients of Chinese medicine in the ZINC 15 [28] (http://zinc.docking.org/) database, used the AutoDock Tool (version 1.5.6, http://autodock.scripps.edu/) to add charges, assign atom types, and confirm rotatable flexible bonds, and used it as the small ligand molecules. Finally, the docking results of active compounds and protein targets were visualized with PyMOL software (version 2.2, https://pymol.org/2/).

## 3. Results

### 3.1. GD-Active Ingredient-Potential Target-Enzyme Network Analysis

**Figure 1:**
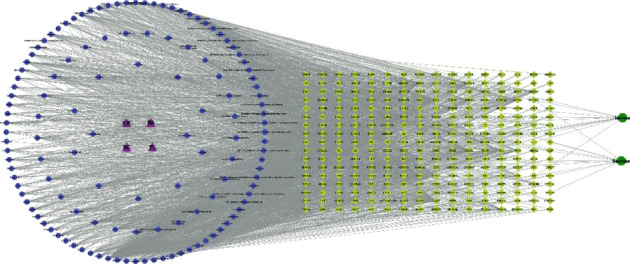
GD-active ingredients-potential targets-enzymes network. The purple triangle represents 4 Chinese herbal medicines, the blue circle represents the active ingredients in GD, the green hexagon represents lactase and sucrase, and the yellow diamond represents the targets.

GD-active ingredient and target information was retrieved from the TCMSP database. There were 146 active ingredients of GD that met OB ≥ 30% and DL ≥ 0.18, including 4 PLR, 36 SR, 14 CR, and 92 GRER. Entering these into the TCMSP database, a total of 2660 targets were found in the search, and 269 targets were obtained after deleting duplicate values. These targets were input into the UniProt database for conversion, and after the final deduplication, only 252 targets successfully found the corresponding gene names. The relevant information of the GD-active ingredients is shown in Table 1. Then, 146 potential active ingredients corresponding to 4 Chinese medicines in GD, 12 lactase targets (e.g., LCT, GLB1, LCTL, MCM6, and HNF1A), and 11 sucrase targets (e.g., LCT, GLB1, LCTL, MCM6, and HNF1A) were input into Cytoscape 3.7.2 software, and the GD-active ingredient-potential target-enzyme network was constructed (Figure 1). The network consisted of 407 nodes and 2,717 edges, among which the purple triangles represented 4 Chinese herbal medicines, the blue circles represented the active ingredients, the green hexagons represented lactase and sucrase, and the yellow diamonds represented the targets. Through the network analysis tool in Cytoscape 3.7.2 software, we screened the active ingredients that mainly played a role according to the “degree” value. The greater the degree value, the more the connection points of the ingredients and the greater the effect. The top active ingredients were quercetin, formononetin, beta-sitosterol, kaempferol, and wogonin. Among them, quercetin is shared by 2 Chinese herbal medicines, with 284 targets including PTGS2, HSP90, CALM, AR, and ESR1, suggesting that the active ingredients may play a key influence in the regulation of lactase and sucrase by GD through the abovementioned targets.

### 3.2. Diarrhea-Potential Target-Enzyme Network Analysis

**Figure 2:**
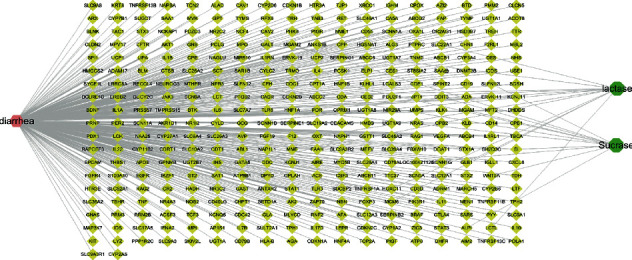
Diarrhea-potential targets-lactase-sucrase network. Pink hexagons represent diseases, green octagons represent lactase and sucrase, and yellow diamonds represent potential targets. The connection between nodes indicates the corresponding relationship between the two.

328 diarrhea-related targets were collected through the DisGeNET database. Then, we constructed the diarrhea-potential target-enzyme network (Figure 2). In this network, there were 347 nodes and 351 connections in the network. Pink hexagons represented diseases, green octagons represented lactase and sucrase, and yellow diamonds represented potential targets. The connection between nodes indicated the corresponding relationship between the two. The more the target points were connected, the more critical role the targets played in the network. The most connected target in the network was HNF1A, indicating that this target might play an important role in diarrhea affecting lactase and sucrase.

### 3.3. GD-Active Ingredient-Enzyme-Diarrhea Network Analysis

**Figure 3:**
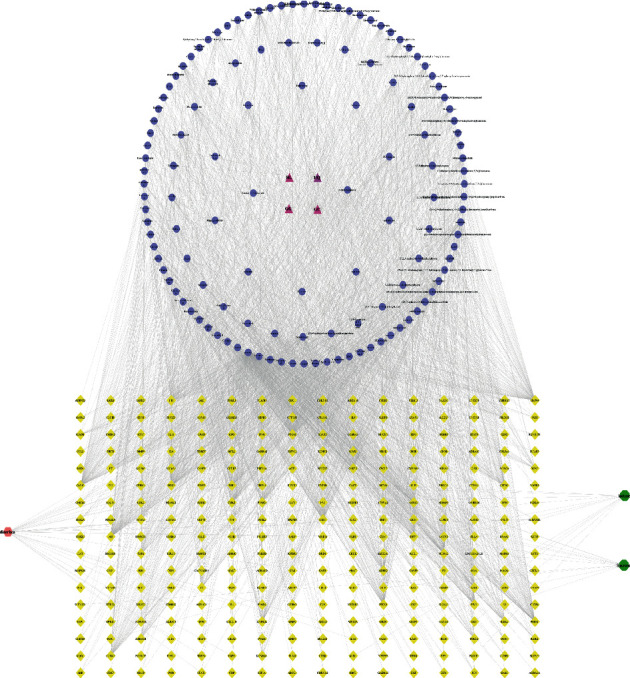
GD-active ingredients-enzymes-diarrhea network. The purple triangle represents 4 Chinese herbal medicines, the blue circle represents the active ingredients in GD, the green polygon represents lactase and sucrase, the pink polygon represents disease, and the yellow diamond represents the targets.

We input the 146 active ingredients of GD and 258 potential targets of diarrhea, as well as the potential targets of lactase and sucrase, into Cytoscape 3.7.2 software to construct the GD-active ingredient-enzyme-diarrhea network (Figure 3). There was a total of 408 nodes and 2748 connections in the network. Purple triangles represented 4 Chinese herbal medicines in GD, blue circles represented active ingredients in GD, pink hexagons represented diseases, green octagons represented lactase and sucrase, and yellow diamonds represented potential targets. From the perspective of ingredients, there were 18 ingredients with a target greater than thirty, of which 6 ingredients were greater than forty. By analyzing the degree centrality (DC), closeness centrality (CC), and betweenness centrality (BC) of the GD-active ingredient-enzyme-diarrhea network, the top five ingredients were quercetin, formononetin, beta-sitosterol, kaempferol, and wogonin, which could be interacted with 284, 76, 74, 58, and 45 target proteins work. As a view of the targets, the top five targets were PTGS2, HSP90, CALM, AR, and ESR1, which interacted with 122, 95, 94, 93, and 92 ingredients (Table 2). Based on these, we found the phenomenon that some ingredients in GD could act on multiple targets, and different ingredients worked together on the same target, which might reflect the mechanism of interaction between multiple compounds and multiple targets in TCM. Through analysis, the ingredients in the network that were closely related to the targets are quercetin, formononetin, beta-sitosterol, kaempferol, and wogonin, indicating that the two active ingredients in GD may be crucial in regulating enzyme activity in the treatment of diarrhea.

### 3.4. Molecular Docking Simulation

**Figure 4:**
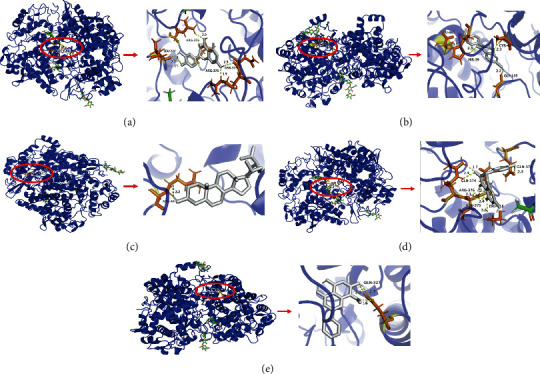
Molecular docking analysis of the potential active ingredients of GD and the potential target. Molecular docking mode of PTGS2 and quercetin, formononetin, *β*-sitosterol kaempferol, and wogonin. (a) PTGS2 acts on quercetin. (b) PTGS2 acts on formononetin. (c) PTGS2 acts on beta-sitosterol. (d) PTGS2 acts on kaempferol. (e) PTGS2 acts on wogonin.

One potential target with five corresponding ingredients was simulated by molecular docking, and the docking results were analyzed. As far as we know, if the value of binding energy is less than 0 kJ/mol, this indicates that the ligand can spontaneously bind to the receptor. The molecular docking results showed that the molecular docking binding energy between the key chemical ingredients and the core target was less than 0 kJ/mol. Among them, formononetin and PTGS2 (−6.8 kcal·mol^−1^), beta-sitosterol and PTGS2 (−5.61 kcal·mol^−1^), and quercetin and PTGS2 (−4.86 kcal·mol^−1^) have better binding energy, from which it can be seen that GD had good affinity and binding activity for core ingredient and key targets (Figure 4).

## 4. Discussion

Intestinal functional enzymes (including lactase and sucrase) are widely distributed in the intestinal mucosa and contents and participated in many important biological metabolic processes and material circulation of the intestinal tract, playing an important role in the digestion, absorption, growth, and development of nutrients in the body [29]. Intestinal microorganisms are the main source of intestinal functional enzymes. While they use the food in the host digestive tract to satisfy their own growth, they can also produce various enzymes, organic acids, and nutrients to assist the host in nutrient absorption and energy metabolism and regulate the balance of intestinal microorganisms, which is closely related to the physiology and pathology of the host [30]. The occurrence of diarrhea reduces the number of physiological bacteria and increases the conditional pathogenic bacteria. Changes in the composition and structure of intestinal microbes will also affect the activity of intestinal enzymes [31]. Some bacteria in the intestines, such as *Bacteroides*, *Streptococcus*, *Lactobacillus*, and *Bifidobacterium*, have excellent enzyme systems, which contribute to their huge catalytic and hydrolysis potential [32]. Lactase and sucrase are important intestinal functional enzymes related to diarrhea. Lactase deficiency or inhibition of its enzyme activity will cause flatulence and even diarrhea [33]. Sucrase deficiency can cause malabsorption syndrome, and adverse symptoms such as diarrhea, bloating, and abdominal pain may occur after eating the sucrose-containing diet [34]. Studies have shown [6, 35] that the activities of lactase and sucrase in the intestinal mucosa and contents of antibiotic-associated diarrhea model mice were reduced. The previous experimental results of the research group found that [21] modeling could increase the total microbial activity in the intestinal contents and intestinal mucosa diarrhea with IDHS mice (*p* < 0.05; *p* < 0.05), suggesting that diarrhea with IDHS caused the increase of the total amount of intestinal microorganism in mice. After modeling, the activities of lactase and sucrase in intestinal contents of model mice were significantly increased (*p* < 0.05; *p* < 0.05), but the activities of lactase and sucrase in the intestinal mucosa were significantly decreased (*p* < 0.05; *p* < 0.05), suggesting that diarrhea with IDHS could cause changes in lactase and sucrase in the intestinal tract of mice.

Most Chinese herbal medicine are taken orally, and the interaction between the drugs and intestinal microbes is often the key to their efficacy. Qiwei Baizhu Powder promoted the recovery of lactase and sucrase activities in antibiotic-associated diarrhea mice [8, 36]. Previous studies indicated [21] that GD markedly reduced the microbial activity in the intestinal contents and intestinal mucosa of mice (*p* < 0.05; *p* < 0.05). The activities of lactase and sucrase in the intestinal contents were significantly decreased (*p* < 0.05; *p* < 0.05), the activities of lactase in the intestinal mucosa decreased (*p* < 0.05), and the activities of sucrase increased (*p* < 0.05). This might be due to the rich content of intestinal flora in intestinal contents, which had a two-way effect on the regulation of lactase and sucrase activities.

Combined with network pharmacological analysis, we constructed the GD-active ingredient-potential target-enzymes network. The analysis results adopted “DC,” “BC,” and “CC” to determine the key nodes and screened the active ingredients and targets according to the network median value ≥ median. The median degree of the active ingredient was 18, and the target degree was 2. Among them, quercetin, formononetin, beta-sitosterol, kaempferol, and wogonin were the main active ingredients screened out in the network, and PTGS2, HSP90, CALM, AR, and ESR1 were the main active targets, and both played an important role in the regulation of lactase and sucrase by GD. At the same time, according to the target information of diarrhea, lactase and sucrase, we constructed the diarrhea-potential target-enzyme network. In this network analysis, we indicated that HNF1A might play an important role in diarrhea affecting lactase and sucrase. Then, we used Cytoscape 3.7.2 software to construct the GD-active ingredient-enzyme-diarrhea network. The results showed that quercetin, formononetin, beta-sitosterol, kaempferol, and wogonin played an important role in the regulation of lactase and sucrase activity by GD in the treatment of diarrhea. Quercetin is one of the more important bioflavonoids found in current research [37, 38]. Quercetin affects the quantity and quality of the intestinal microbiota, thereby indirectly affecting their own metabolism and bioavailability [39]. Studies have indicated that quercetin can enhance the tolerance of diabetic animals to hyperglycemia and control the further development of diabetic complications by regulating the activity of lactase in the intestine of diabetic rats [40]. According to the results of in vitro experiments [41], quercetin significantly support the proliferation of *Lactobacillus* within a certain concentration range, and *Lactobacillus* contributes to the hydrolysis of intestinal enzymes. Some studies have reported that active ingredients such as formononetin and *β*-sitosterol have certain anti-inflammatory effects. These anti-inflammatory effects can be achieved through the regulation of intestinal flora. Among them, formononetin achieve the anti-inflammatory effect by regulating intestinal flora in the process of diarrhea [42]. *β*-Sitosterol treatment to intestinal epithelial cells significantly increases expression of antimicrobial peptides and reduces survival of intracellular *Salmonella typhimurium* [43]. Also, some bacteria in the intestine are involved in affecting the regulation of intestinal enzymes. It is inferred that these active ingredients may regulate intestinal enzyme activity by regulating some bacteria in the intestine, thus achieving the effect of treating diarrhea. On this basis, we also found that some ingredients in GD might act on multiple targets, while the phenomenon that different ingredients act on the same target together reflected the mechanism of interaction between multiple ingredients in TCM and multiple targets.

Molecular docking studies were carried out to confirm the interaction between active ingredients and diarrhea-related potential target at the molecular level. This determined the binding pose of the active ingredients when bound to the target protein and suggested that the active ingredients might have high binding affinity for proteins encoded by diarrhea-related genes. The results of this study showed that the combination of GD and diarrhea target binding energy was negative, indicating that there was binding activity between the ingredient and the target protein. Among these interactions, the most important node (formononetin) had the high affinity with potential target in the molecular docking score. This result indicated that instead of one ingredient interacting with one target protein, one or more ingredients interact with multiple target proteins. Based on this, it was speculated that these ingredients might play an important role in GD in regulating the expression of diarrhea target protein.

## 5. Conclusions

To sum up, the active ingredients in GD (such as quercetin, formononetin, and *β*-sitosterol) may enhance the microbial activity of the intestinal mucosa of diarrhea with IDHS mice and decrease the intestinal contents through multiple targets, so as to achieve the therapeutic effect of diarrhea, in order to provide the basis for the clinical diarrhea differentiation diagnosis and the mechanism research of the curative effect of TCM prescription. Simultaneously, there were still limitations in this study, i.e., the forecast results were obtained based on existing limited databases and might involve the existence of factors such as insufficient research on key targets of diarrhea, unknown chemical composition and structure of GD, and the interaction relationship between the ingredients. Additionally, this study only provides a preliminary hypothesis for the scientific study of TCM; animal experiments, molecular experiments, and clinical investigations should be performed to verify the mechanism of GD against diarrhea in future studies.

## Figures and Tables

**Table 1:** Active ingredients of GD.

Chinese medicine	Mol ID	Molecule Name	OB (%)	DL
*Puerarie Lobatae Radix*	MOL000392	Formononetin	69.67	0.21
*Puerarie Lobatae Radix*	MOL000358	beta-Sitosterol	36.91	0.75
*Puerarie Lobatae Radix*	MOL002959	3′-Methoxydaidzein	48.57	0.24
*Puerarie Lobatae Radix*	MOL003629	Daidzein-4,7-diglucoside	47.27	0.67
*Scutellariae Radix*	MOL002934	Neobaicalein	104.34	0.44
*Scutellariae Radix*	MOL002932	Panicolin	76.26	0.29
*Scutellariae Radix*	MOL012246	5,7,4′-Trihydroxy-8-methoxyflavanone	74.24	0.26
*Scutellariae Radix*	MOL002927	Skullcapflavone II	69.51	0.44
*Scutellariae Radix*	MOL002911	2,6,2′,4′-Tetrahydroxy-6′-methoxychaleone	69.04	0.22
*Scutellariae Radix*	MOL002937	Dihydrooroxylin	66.06	0.23
*Scutellariae Radix*	MOL000228	(2R)-7-hydroxy-5-methoxy-2-phenylchroman-4-one	55.23	0.2
*Scutellariae Radix*	MOL002915	Salvigenin	49.07	0.33
*Scutellariae Radix*	MOL000073	ent-Epicatechin	48.96	0.24
*Scutellariae Radix*	MOL002917	5,2′,6′-Trihydroxy-7,8-dimethoxyflavone	45.05	0.33
*Scutellariae Radix*	MOL008206	Moslosooflavone	44.09	0.25
*Scutellariae Radix*	MOL000449	Stigmasterol	43.83	0.76
*Scutellariae Radix*	MOL001490	bis[(2S)-2-ethylhexyl] benzene-1,2-dicarboxylate	43.59	0.35
*Scutellariae Radix*	MOL002879	Diop	43.59	0.39
*Scutellariae Radix*	MOL002897	Epiberberine	43.09	0.78
*Scutellariae Radix*	MOL002928	Oroxylin a	41.37	0.23
*Scutellariae Radix*	MOL002914	Eriodyctiol (flavanone)	41.35	0.24
*Scutellariae Radix*	MOL002910	Carthamidin	41.15	0.24
*Scutellariae Radix*	MOL002913	Dihydrobaicalin_qt	40.04	0.21
*Scutellariae Radix*	MOL000525	Norwogonin	39.4	0.21
*Scutellariae Radix*	MOL010415	11,13-Eicosadienoic acid, methyl ester	39.28	0.23
*Scutellariae Radix*	MOL002926	Dihydrooroxylin A	38.72	0.23
*Scutellariae Radix*	MOL012266	Rivularin	37.94	0.37
*Scutellariae Radix*	MOL002908	5,8,2′-Trihydroxy-7-methoxyflavone	37.01	0.27
*Scutellariae Radix*	MOL002925	5,7,2′,6′-Tetrahydroxyflavone	37.01	0.24
*Scutellariae Radix*	MOL000358	beta-Sitosterol	36.91	0.75
*Scutellariae Radix*	MOL000359	Sitosterol	36.91	0.75
*Scutellariae Radix*	MOL012245	5,7,4′-Trihydroxy-6-methoxyflavanone	36.63	0.27
*Scutellariae Radix*	MOL002933	5,7,4′-Trihydroxy-8-methoxyflavone	36.56	0.27
*Scutellariae Radix*	MOL001689	Acacetin	34.97	0.24
*Scutellariae Radix*	MOL002909	5,7,2,5-Tetrahydroxy-8,6-dimethoxyflavone	33.82	0.45
*Scutellariae Radix*	MOL001506	Supraene	33.55	0.42
*Scutellariae Radix*	MOL002714	Baicalein	33.52	0.21
*Scutellariae Radix*	MOL000552	5,2′-Dihydroxy-6,7,8-trimethoxyflavone	31.71	0.35
*Scutellariae Radix*	MOL000173	Wogonin	30.68	0.23
*Scutellariae Radix*	MOL001458	Coptisine	30.67	0.86
*Coptidis Rhizoma*	MOL002907	Corchoroside A_qt	104.95	0.78
*Coptidis Rhizoma*	MOL008647	Moupinamide	86.71	0.26
*Coptidis Rhizoma*	MOL000785	Palmatine	64.6	0.65
*Coptidis Rhizoma*	MOL000622	Magnograndiolide	63.71	0.19
*Coptidis Rhizoma*	MOL002903	(R)-canadine	55.37	0.77
*Coptidis Rhizoma*	MOL000098	Quercetin	46.43	0.28
*Coptidis Rhizoma*	MOL002668	Worenine	45.83	0.87
*Coptidis Rhizoma*	MOL013352	Obacunone	43.29	0.77
*Coptidis Rhizoma*	MOL002897	Epiberberine	43.09	0.78
*Coptidis Rhizoma*	MOL001454	Berberine	36.86	0.78
*Coptidis Rhizoma*	MOL002904	Berlambine	36.68	0.82
*Coptidis Rhizoma*	MOL002894	Berberrubine	35.74	0.73
*Coptidis Rhizoma*	MOL000762	Palmidin A	35.36	0.65
*Coptidis Rhizoma*	MOL001458	Coptisine	30.67	0.86
*Glycyrrhiza Radix Et Rhizoma*	MOL002311	Glycyrol	90.78	0.67
*Glycyrrhiza Radix Et Rhizoma*	MOL004990	7,2′,4′-Trihydroxy-5-methoxy-3-arylcoumarin	83.71	0.27
*Glycyrrhiza Radix Et Rhizoma*	MOL004904	Licopyranocoumarin	80.36	0.65
*Glycyrrhiza Radix Et Rhizoma*	MOL004891	Shinpterocarpin	80.3	0.73
*Glycyrrhiza Radix Et Rhizoma*	MOL005017	Phaseol	78.77	0.58
*Glycyrrhiza Radix Et Rhizoma*	MOL004841	Licochalcone B	76.76	0.19
*Glycyrrhiza Radix Et Rhizoma*	MOL004810	Glyasperin F	75.84	0.54
*Glycyrrhiza Radix Et Rhizoma*	MOL001484	Inermine	75.18	0.54
*Glycyrrhiza Radix Et Rhizoma*	MOL000500	Vestitol	74.66	0.21
*Glycyrrhiza Radix Et Rhizoma*	MOL005007	Glyasperins M	72.67	0.59
*Glycyrrhiza Radix Et Rhizoma*	MOL004941	(2R)-7-hydroxy-2-(4-hydroxyphenyl)chroman-4-one	71.12	0.18
*Glycyrrhiza Radix Et Rhizoma*	MOL004959	1-Methoxyphaseollidin	69.98	0.64
*Glycyrrhiza Radix Et Rhizoma*	MOL000392	Formononetin	69.67	0.21
*Glycyrrhiza Radix Et Rhizoma*	MOL004863	3-(3,4-Dihydroxyphenyl)-5,7-dihydroxy-8-(3-methylbut-2-enyl)chromone	66.37	0.41
*Glycyrrhiza Radix Et Rhizoma*	MOL004903	Liquiritin	65.69	0.74
*Glycyrrhiza Radix Et Rhizoma*	MOL004808	Glyasperin B	65.22	0.44
*Glycyrrhiza Radix Et Rhizoma*	MOL004829	Glepidotin B	64.46	0.34
*Glycyrrhiza Radix Et Rhizoma*	MOL004855	Licoricone	63.58	0.47
*Glycyrrhiza Radix Et Rhizoma*	MOL004914	1,3-Dihydroxy-8,9-dimethoxy-6-benzofurano [3,2-c]chromenone	62.9	0.53
*Glycyrrhiza Radix Et Rhizoma*	MOL004835	Glypallichalcone	61.6	0.19
*Glycyrrhiza Radix Et Rhizoma*	MOL004907	Glyzaglabrin	61.07	0.35
*Glycyrrhiza Radix Et Rhizoma*	MOL005000	Gancaonin G	60.44	0.39
*Glycyrrhiza Radix Et Rhizoma*	MOL004824	(2S)-6-(2,4-dihydroxyphenyl)-2-(2-hydroxypropan-2-yl)-4-methoxy-2,3-dihydrofuro[3,2-g]chromen-7-one	60.25	0.63
*Glycyrrhiza Radix Et Rhizoma*	MOL004849	3-(2,4-Dihydroxyphenyl)-8-(1,1-dimethylprop-2-enyl)-7-hydroxy-5-methoxy-coumarin	59.62	0.43
*Glycyrrhiza Radix Et Rhizoma*	MOL004328	Naringenin	59.29	0.21
*Glycyrrhiza Radix Et Rhizoma*	MOL005003	Licoagrocarpin	58.81	0.58
*Glycyrrhiza Radix Et Rhizoma*	MOL004838	8-(6-Hydroxy-2-benzofuranyl)-2,2-dimethyl-5-chromenol	58.44	0.38
*Glycyrrhiza Radix Et Rhizoma*	MOL005012	Licoagroisoflavone	57.28	0.49
*Glycyrrhiza Radix Et Rhizoma*	MOL000211	Mairin	55.38	0.78
*Glycyrrhiza Radix Et Rhizoma*	MOL005018	Xambioona	54.85	0.87
*Glycyrrhiza Radix Et Rhizoma*	MOL005020	Dehydroglyasperins C	53.82	0.37
*Glycyrrhiza Radix Et Rhizoma*	MOL004993	8-Prenylated eriodictyol	53.79	0.4
*Glycyrrhiza Radix Et Rhizoma*	MOL004908	Glabridin	53.25	0.47
*Glycyrrhiza Radix Et Rhizoma*	MOL004910	Glabranin	52.9	0.31
*Glycyrrhiza Radix Et Rhizoma*	MOL004879	Glycyrin	52.61	0.47
*Glycyrrhiza Radix Et Rhizoma*	MOL004912	Glabrone	52.51	0.5
*Glycyrrhiza Radix Et Rhizoma*	MOL004885	Licoisoflavanone	52.47	0.54
*Glycyrrhiza Radix Et Rhizoma*	MOL003656	Lupiwighteone	51.64	0.37
*Glycyrrhiza Radix Et Rhizoma*	MOL004856	Gancaonin A	51.08	0.4
*Glycyrrhiza Radix Et Rhizoma*	MOL000239	Jaranol	50.83	0.29
*Glycyrrhiza Radix Et Rhizoma*	MOL004820	Kanzonols W	50.48	0.52
*Glycyrrhiza Radix Et Rhizoma*	MOL005001	Gancaonin H	50.1	0.78
*Glycyrrhiza Radix Et Rhizoma*	MOL005016	Odoratin	49.95	0.3
*Glycyrrhiza Radix Et Rhizoma*	MOL000354	Isorhamnetin	49.6	0.31
*Glycyrrhiza Radix Et Rhizoma*	MOL004848	Licochalcone G	49.25	0.32
*Glycyrrhiza Radix Et Rhizoma*	MOL002565	Medicarpin	49.22	0.34
*Glycyrrhiza Radix Et Rhizoma*	MOL004857	Gancaonin B	48.79	0.45
*Glycyrrhiza Radix Et Rhizoma*	MOL004827	Semilicoisoflavone B	48.78	0.55
*Glycyrrhiza Radix Et Rhizoma*	MOL004913	1,3-Dihydroxy-9-methoxy-6-benzofurano [3,2-c]chromenone	48.14	0.43
*Glycyrrhiza Radix Et Rhizoma*	MOL000417	Calycosin	47.75	0.24
*Glycyrrhiza Radix Et Rhizoma*	MOL004961	Quercetin der.	46.45	0.33
*Glycyrrhiza Radix Et Rhizoma*	MOL000098	Quercetin	46.43	0.28
*Glycyrrhiza Radix Et Rhizoma*	MOL004911	Glabrene	46.27	0.44
*Glycyrrhiza Radix Et Rhizoma*	MOL004898	(E)-3-[3,4-dihydroxy-5-(3-methylbut-2-enyl)phenyl]-1-(2,4-dihydroxyphenyl)prop-2-en-1-one	46.27	0.31
*Glycyrrhiza Radix Et Rhizoma*	MOL004974	3′-Methoxyglabridin	46.16	0.57
*Glycyrrhiza Radix Et Rhizoma*	MOL004811	Glyasperin C	45.56	0.4
*Glycyrrhiza Radix Et Rhizoma*	MOL004949	Isolicoflavonol	45.17	0.42
*Glycyrrhiza Radix Et Rhizoma*	MOL004828	Glepidotin A	44.72	0.35
*Glycyrrhiza Radix Et Rhizoma*	MOL004948	Isoglycyrol	44.7	0.84
*Glycyrrhiza Radix Et Rhizoma*	MOL004866	2-(3,4-Dihydroxyphenyl)-5,7-dihydroxy-6-(3-methylbut-2-enyl)chromone	44.15	0.41
*Glycyrrhiza Radix Et Rhizoma*	MOL004966	3′-Hydroxy-4′-O-methylglabridin	43.71	0.57
*Glycyrrhiza Radix Et Rhizoma*	MOL004915	Eurycarpin A	43.28	0.37
*Glycyrrhiza Radix Et Rhizoma*	MOL003896	7-Methoxy-2-methyl isoflavone	42.56	0.2
*Glycyrrhiza Radix Et Rhizoma*	MOL000422	Kaempferol	41.88	0.24
*Glycyrrhiza Radix Et Rhizoma*	MOL004883	Licoisoflavone	41.61	0.42
*Glycyrrhiza Radix Et Rhizoma*	MOL005008	Glycyrrhiza flavonol A	41.28	0.6
*Glycyrrhiza Radix Et Rhizoma*	MOL005013	18*α*-Hydroxyglycyrrhetic acid	41.16	0.71
*Glycyrrhiza Radix Et Rhizoma*	MOL004924	(−)-Medicocarpin	40.99	0.95
*Glycyrrhiza Radix Et Rhizoma*	MOL000497	Licochalcone a	40.79	0.29
*Glycyrrhiza Radix Et Rhizoma*	MOL004980	Inflacoumarin A	39.71	0.33
*Glycyrrhiza Radix Et Rhizoma*	MOL004815	(E)-1-(2,4-dihydroxyphenyl)-3-(2,2-dimethylchromen-6-yl)prop-2-en-1-one	39.62	0.35
*Glycyrrhiza Radix Et Rhizoma*	MOL004989	6-Prenylated eriodictyol	39.22	0.41
*Glycyrrhiza Radix Et Rhizoma*	MOL004884	Licoisoflavone B	38.93	0.55
*Glycyrrhiza Radix Et Rhizoma*	MOL004991	7-Acetoxy-2-methylisoflavone	38.92	0.26
*Glycyrrhiza Radix Et Rhizoma*	MOL004957	HMO	38.37	0.21
*Glycyrrhiza Radix Et Rhizoma*	MOL004917	Glycyroside	37.25	0.79
*Glycyrrhiza Radix Et Rhizoma*	MOL000359	Sitosterol	36.91	0.75
*Glycyrrhiza Radix Et Rhizoma*	MOL004945	(2S)-7-hydroxy-2-(4-hydroxyphenyl)-8-(3-methylbut-2-enyl)chroman-4-one	36.57	0.32
*Glycyrrhiza Radix Et Rhizoma*	MOL004978	2-[(3R)-8,8-dimethyl-3,4-dihydro-2H-pyrano [6,5-f]chromen-3-yl]-5-methoxyphenol	36.21	0.52
*Glycyrrhiza Radix Et Rhizoma*	MOL004935	Sigmoidin-B	34.88	0.41
*Glycyrrhiza Radix Et Rhizoma*	MOL004905	3,22-Dihydroxy-11-oxo-delta(12)-oleanene-27-alpha-methoxycarbonyl-29-oic acid	34.32	0.55
*Glycyrrhiza Radix Et Rhizoma*	MOL004882	Licocoumarone	33.21	0.36
*Glycyrrhiza Radix Et Rhizoma*	MOL004860	Licorice glycoside E	32.89	0.27
*Glycyrrhiza Radix Et Rhizoma*	MOL001792	DFV	32.76	0.18
*Glycyrrhiza Radix Et Rhizoma*	MOL004988	Kanzonol F	32.47	0.89
*Glycyrrhiza Radix Et Rhizoma*	MOL004833	Phaseolinisoflavan	32.01	0.45
*Glycyrrhiza Radix Et Rhizoma*	MOL004814	Isotrifoliol	31.94	0.42
*Glycyrrhiza Radix Et Rhizoma*	MOL004805	(2S)-2-[4-hydroxy-3-(3-methylbut-2-enyl)phenyl]-8,8-dimethyl-2,3-dihydropyrano [2,3-f]chromen-4-one	31.79	0.72
*Glycyrrhiza Radix Et Rhizoma*	MOL004985	Icos-5-enoic acid	30.7	0.2
*Glycyrrhiza Radix Et Rhizoma*	MOL004996	Gadelaidic acid	30.7	0.2
*Glycyrrhiza Radix Et Rhizoma*	MOL004864	5,7-Dihydroxy-3-(4-methoxyphenyl)-8-(3-methylbut-2-enyl)chromone	30.49	0.41
*Glycyrrhiza Radix Et Rhizoma*	MOL004806	Euchrenone	30.29	0.57

*Note.* OB: oral bioavailability; DL: drug-likeness.

**Table 2:** Top 30 active ingredients or potential target proteins in the GD-active ingredient-enzyme-diarrhea network.

Name	DC	BC	CC
Quercetin	284	0.4742	0.4963
PTGS2	122	0.0911	0.5218
HSP90	95	0.0595	0.4845
CALM	94	0.0230	0.3818
AR	93	0.0352	0.4532
ESR1	92	0.0205	0.3602
NOS2	91	0.0187	0.3769
GC	88	0.0512	0.4553
PRSS1	79	0.0263	0.4386
Formononetin	76	0.0389	0.3741
PTGS1	76	0.0494	0.4678
beta-Sitosterol	74	0.0366	0.3755
PPARG	70	0.0276	0.4386
SCN5A	69	0.0319	0.4266
NCOA2	68	0.0367	0.4248
CDK2	66	0.0065	0.3342
PIM1	65	0.0050	0.3215
F10	63	0.0159	0.4006
ESR2	63	0.0049	0.3210
GSK3B	62	0.0052	0.3282
Kaempferol	58	0.0635	0.3884
CHEK1	57	0.0049	0.3272
CCNA2	55	0.0035	0.3160
DPP4	52	0.0172	0.4136
MAPK14	52	0.0037	0.3230
Wogonin	45	0.0437	0.3769
ADRB2	45	0.0184	0.4095
RXRA	45	0.0150	0.4014
7-Methoxy-2-methyl isoflavone	43	0.0178	0.3783
F2	41	0.0093	0.3884

*Note.* DC: degree centrality; BC: betweenness centrality; CC: closeness centrality.

## Data Availability

The data used to support the findings of this study are available from the corresponding author upon request.

## Abbreviations

IDHS: Intestinal damp-heat syndrome
FDA: Fluorescein diacetate
GD: Gegenqinlian decoction
PLR: *Puerarie Lobatae Radix*
SR: *Scutellariae Radix*
CR: *Coptidis Rhizoma*
GRER: *Glycyrrhiza Radix Et Rhizoma*
TCM: Traditional Chinese medicine
TCMSP: Traditional Chinese Medicine Systems Pharmacology
OB: Oral bioavailability
DL: Drug-likeness
UniProt: Universal protein
DC: Degree centrality
CC: Closeness centrality
BC: Betweenness centrality
NCBI: National Center for Biotechnology Information.

## References

[1] Zhang S. S., Wang C. J., Li Y. F., Wang N. (2017). Expert consensus on diagnosis and treatment of diarrhea in Chinese medicine. *Journal of Traditional Chinese Medical Sciences*.

[2] Zhao Y., Zhao Y. F., Peng J. H., Feng Q., Hu Y. Y. (2019). Thoughts on the relationship between damp-heat syndrome of chronic liver disease and intestinal microecology. *World Chinese Medicine*.

[3] Ding P. F., Li J. X., Guo Y., Mao T. Y., Zhao X. J. (2018). Study on diversity of intestinal flora of patients with large damp heat syndrome and ulcerative colitis by high-throughput sequencing. *World Science and Technology-Modernization of Traditional Chinese Medicine*.

[4] Wark G., Samocha-Bonet D., Ghaly S., Danta M. (2020). The role of diet in the pathogenesis and management of inflammatory bowel disease: a review. *Nutrients*.

[5] Zhoujin T., Hai W., Fulin L. (2012). Effect of ultra-micro powder qiweibaishusan on the intestinal microbiota and enzyme activities in mice. *Acta Ecologica Sinica*.

[6] Hui H. Y., He L., Peng X. X. (2017). The influence of antibiotics modeling on the intestinal lactase activity in dysbacteriotic diarrhea mice. *Chinese Journal of Preventive Veterinary Medicine*.

[7] Hu R., Zhang M. T., Tang F. (2010). Effect of Weichang’an pill on intestinal digestive ferment and the AQP4 concentration in proximal colon in IBS-D rats. *China Journal of Chinese Materia Medica*.

[8] Guo K. X., Peng X. X., Mao Y. N. (2019). Effect of qiwei baizhu san on intestinal sucrase activity in mice with diarrhea. *Chinese Journal of Microecology*.

[9] Xu Y. X., He L. L., Liu Y. X. (2019). Effects of biochar addition on enzyme activity and fertility in paddy soil after six years. *Chinese Journal of Applied Ecology*.

[10] Tang Y., Wu Y., Hui H. Y., Tan Z. J. (2020). Effect of Tongxieyaofang prescription on intestinal microbial activity in mice with Ganqichenpi diarrhea. *Chinese Journal of Microecology*.

[11] Swisher R., Carroll G. C. (1980). Fluorescein diacetate hydrolysis as an estimator of microbial biomass on coniferous needle surfaces. *Microbial Ecology*.

[12] Bian Z. X., Qin H. Y., Tian S. L., Qi S. D. (2011). Combined effect of early life stress and acute stress on colonic sensory and motor responses through serotonin pathways: differences between proximal and distal colon in rats. *Stress (Amsterdam, Netherlands)*.

[13] Xiong X. J. (2019). Gegen Qinlian Decoction formula syndrome and its application for diabetes, hypertention, hyperlipidemia, and obesity. *China Journal of Chinese Materia Medica*.

[14] Li H. Y., Zhao L. H., Zhang B. (2014). A network pharmacology approach to determine active compounds and action mechanisms of ge-gen-qin-lian decoction for treatment of type 2 diabetes. *Evidence-Based Complementary and Alternative Medicine*.

[15] Feng X. G., Yan Y. Z., Zeng Y. P., Guo Y. F. (2016). The effect of gegen qianlian decoction on intestinal flora damp-heat syndrome of type 2 diabetes. *World Journal of integrated Traditional and Western Medicine*.

[16] Chen Y., Lu J., Zhu S. M. (2019). Effect of gegen qinlian decoction and it’s different compatibility groups on gut microbiota in rats with acute enteritis based on high-throughput sequencing. *Chinese Journal of Traditional Chinese Medicine*.

[17] Shi X. G., Shi J. X., Liu H. T. (2018). Effects of buzhong yiqi decotion (BZYQ) on SGLT1/NHE3 pathway in rats with spleen deficient diarrhea. *Traditional Chinese Medicine and Clinical Pharmacology*.

[18] He Y. S., Tang Y., Xiao Y. F., Xiao N. Q., Hui H. Y. (2020). Effects of gegen qinlian decoction on microbial growth in simulated gastric and intestinal liquid. *Chinese Journal of Microecology*.

[19] Liu J., Li Y., Zhang Y. (2019). A network pharmacology approach to explore the mechanisms of qishen granules in heart failure. *Medical Science Monitor*.

[20] Li S., Zhang B. (2013). Traditional chinese medicine network pharmacology: theory, methodology and application. *Chinese Journal of Natural Medicines*.

[21] Hui H. Y., He Y. S., Luo Y. C., Tan Z. J. (2020). Effect of ge-gen-qin-lian decoction on intestinal microbial activity and enzyme activity in diarrhea mice with intestinal dampness-heat syndrome. *Chinese Journal of Applied & Environmental Biology*.

[22] Ru J., Li P., Wang J. (2014). TCMSP: a database of systems pharmacology for drug discovery from herbal medicines. *Journal of Cheminformatics*.

[23] Liu L. P., Long X., Cao X. S., Xu X. Y., Luo Y. W., Gui R. (2020). Research on active compounds of maxingyigan decoction for treatment of coronavirus disease 2019 based on network pharmacology and molecular docking. *Chinese Traditional and Herbal Drugs*.

[24] Guo X., Ji J., Feng Z., Hou X., Luo Y., Mei Z. (2020). A network pharmacology approach to explore the potential targets underlying the effect of sinomenine on rheumatoid arthritis. *International Immunopharmacology*.

[25] Du X., Zhao L., Yang Y. (2020). Investigation of the mechanism of action of *Porana sinensis* hemsl. against gout arthritis using network pharmacology and experimental validation. *Journal of Ethnopharmacology*.

[26] Franz M., Lopes C. T., Huck G. (2016). A graph theory library for visualisation and analysis. *Bioinformatics*.

[27] Goodsell D. S., Zardecki C., Di Costanzo L. (2020). RCSB protein data bank: enabling biomedical research and drug discovery. *Protein Science*.

[28] Sterling T., Irwin J. J. (2015). Zinc 15-ligand discovery for everyone. *Journal of Chemical Information and Modeling*.

[29] Chen J. F., Qu X. Y. (2020). Effects of montmorillonite and *Bacillus subtilis* on plasma biochemical indexes, small intestinal brush edge enzyme activity and mucin expression in layers. *Chinese Journal of Animal Science*.

[30] He Y. S., Tan Z. J., Li D. D., Hui H. Y. (2019). Effect of bao-he pills on intestinal microorganisms and enzyme activity in mice with dyspepsia. *Chinese Journal of Microecology*.

[31] Guo S. J., Jiang D. J., Li Z. L., Zhang Q., Zhang L. (2018). Research progress on relationship between intestinal flora and common gastrointestinal diseases and treatments of Chinese materia medica and microecological preparations. *Chinese Traditional and Herbal Drugs*.

[32] Long C.-X., Guo Y.-F., Liu Y.-W., Peng X.-X., Tan Z.-J. (2017). Immunoprotective effect of traditional chinese medicine on intestinal mucosa. *World Chinese Journal of Digestology*.

[33] He L., Long C. X., Liu Y. J., Hui H. Y., Tan Z. J. (2017). Research progress on microorganism lactase. *Journal of the Food and Fermentation Industry*.

[34] Neyrinck A. M., Pachikian B., Taminiau B. (2016). Intestinal sucrase as a novel target contributing to the regulation of glycemia by prebiotics. *PLoS One*.

[35] Hui H. Y., Peng M. J., Xiao N. Q., Li D. D. (2018). The effect of modeling dysbacterial diarrhea with antibiotics on molecular diversity of intestinal microbiota in mice. *Chinese Journal of Microecology*.

[36] Hui H. Y., Shen K. J., Li D. D., Tan Z. J. (2018). Influence of qiwei baizhu powder on the lactase activity in intestine of mice with diarrhea induced by antibiotics. *Chinese Journal of Microecology*.

[37] Shamala S., Baskaran G., Noor A. S., Siti A. A., Mohd Y. S., Senthil K. (2014). Antiartherosclerotic effects of plant flavonoids. *BioMed Research International*.

[38] Renu N. J. (2017). Antibacterial properties of quercetin. *Microbiological Research*.

[39] Murota K., Nakamura Y., Uehara M. (2018). Flavonoid metabolism: the interaction of metabolites and gut microbiota. *Bioscience, Biotechnology, and Biochemistry*.

[40] Ramachandra R., Shetty A. K., Salimath P. V. (2005). Quercetin alleviates activities of intestinal and renal disaccharidases in streptozotocin-induced diabetic rats. *Molecular Nutrition & Food Research*.

[41] Chen Y. M., Hu M. J., Zeng Y. P. (2010). Study on the regulation of quercetin on intestinal flora. *Food Research and Development*.

[42] Jin X., Lu Y., Chen S., Chen D. (2020). UPLC-MS identification and anticomplement activity of the metabolites of *Sophora tonkinensis* flavonoids treated with human intestinal bacteria. *Journal of Pharmaceutical and Biomedical Analysis*.

[43] Ding K., Tan Y. Y., Ding Y. (2019). *β*‐Sitosterol improves experimental colitis in mice with a target against pathogenic bacteria. *Journal of Cellular Biochemistry*.

